# Dosage reduction of low weight heparin in patients with renal dysfunction: Effects on anti-Xa levels and clinical outcomes

**DOI:** 10.1371/journal.pone.0239222

**Published:** 2020-10-01

**Authors:** Paul Hornung, Meriem Khairoun, Friedo W. Dekker, Karin A. H. Kaasjager, Albert Huisman, Lily Jakulj, Willem Jan W. Bos, Frits R. Rosendaal, Marianne C. Verhaar, Gurbey Ocak

**Affiliations:** 1 Department of Nephrology and Hypertension, University Medical Center Utrecht, Utrecht, the Netherlands; 2 Department of Clinical Epidemiology, Leiden University Medical Center, Leiden, the Netherlands; 3 Department of Internal Medicine, University Medical Center Utrecht, Utrecht, the Netherlands; 4 Department of Clinical Chemistry and Haematology, University Medical Center Utrecht, Utrecht, the Netherlands; 5 Dianet Dialysis Center and Department of Nephrology, Amsterdam University Medical Center, Amsterdam, the Netherlands; 6 Department of Internal Medicine, St Antonius Hospital, Nieuwegein, the Netherlands; 7 Department of Internal Medicine, Leiden University Medical Center, Leiden, the Netherlands; Istituto Di Ricerche Farmacologiche Mario Negri, ITALY

## Abstract

**Background:**

To prevent bio-accumulation of low molecular weight heparins (LMWHs) in patients with decreased kidney function, dosage reduction and anti-Xa monitoring has been suggested. The aim of this study was to investigate the effect of pre-emptive dosage reduction of LMWH on anti-Xa levels. Furthermore, we investigated the association between anti-Xa levels and bleeding, thrombotic events and mortality.

**Methods:**

In this single center study, we followed 499 patients with decreased renal function in whom anti-Xa levels were measured. We observed how many patients had anti-Xa levels that fell within the reference range, with a standard protocol of a pre-emptive dosage reduction of LMWH (25% reduction in patients with an estimated glomerular filtration rate (eGFR) between 30 and 60 ml/min/1.73m^2^ and a reduction of 50% in patients with an eGFR below the 30 ml/min/1.73m^2^). Furthermore, Cox proportional hazard analyses were used to estimate hazard ratios to investigate the association between anti-Xa levels and major bleeding, thrombotic events and mortality within three months of follow-up.

**Results:**

In a cohort of 499 patients (445 dalteparin and 54 nadroparin users), a pre-emptive dosage reduction of LMWH led to adequate levels of anti-Xa in only 19% of the patients (12% for the dalteparin users and 50% for nadroparin users). We did not find an association between anti-Xa levels and bleeding, thrombosis or mortality.

**Conclusion:**

Pre-emptive dosage reduction of LMWH leads to low anti-Xa levels in a large proportion, but this was not associated with bleeding, thrombosis or mortality.

## Introduction

Low molecular weight heparins (LMWHs) are derived from unfractionated heparin by depolymerization and have superior pharmacokinetic properties as compared with unfractionated heparin [1–6]. The main anticoagulant pathway of LMWH is through inhibition of activated factor X (factor Xa), which can be monitored by anti-Xa levels in blood [1]. However, monitoring of anti-Xa levels is not routinely performed.

The predominant clearance of LMWH is by the kidneys, and consequently, the biologic half-life is prolonged in patients with impaired renal function [7, 8]. Several studies found increased bleeding risks in patients with renal failure who used LMWHs [9–11]. In a meta-analysis of twelve studies involving 4,971 patients who used LMWHs, patients with an estimated glomerular filtration rate (eGFR) ≤30 ml/min/1.73m^2^ had a 2.3-fold increased major bleeding risk as compared with patients with an eGFR>30 ml/min/1.73m^2^ [9].

In order to prevent bio-accumulation of LMWHs, the American College of Chest Physicians Evidence-Based Clinical Practice Guidelines on antithrombotic therapy suggest a dosage reduction of LMWHs combined with anti-Xa monitoring in patients with decreased renal function [12]. Furthermore, the Dutch Federation of Nephrology guideline for anticoagulation with LMWHs in patients with renal impairment recommends to reduce the dose of LMWHs by 25% in patients with an eGFR between the 30 and 60 ml/min/1.73m^2^ and to decrease the dose of LMWHs by 50% in patients with an eGFR≤30 ml/min/1.73m^2^ [13]. In addition, anti-Xa monitoring is recommended in patients with an eGFR<60 ml/min/1.73m^2^ with advised peak target ranges of 1.0–2.0 U/ml for once daily dosing and 0.6–1.0 U/ml for twice daily dosing [13].

Although advised in current guidelines, it is not known whether this dosage reduction of LMWHs in patients with an eGFR<60 ml/min/1.73m^2^ leads to adequate anti-Xa levels. In addition, it is unknown whether the reduced dosage is still effective with regard to clinical endpoints. Therefore, anti-Xa monitoring for safety purposes is still a matter of debate.

The aim of this study was to investigate whether pre-emptive dosage reduction of LMWHs (nadroparin and dalteparin) leads to adequate anti-Xa levels. Furthermore, we aimed to investigate the association between anti-Xa levels and clinical outcomes (major bleeding, thrombotic events and mortality).

## Methods

### Study design and population

This single center cohort study was conducted at the University Medical Center Utrecht, the Netherlands. Adult patients ≥18 years who were admitted to the Department of the Internal Medicine between January 2012 and January 2019 and in whom an anti-Xa measurement was measured, were eligible for this study. We only included patients who used dalteparin or nadroparin in our study with a therapeutic dosage of LMWH and an eGFR below the 60 ml/min/1.73m^2^. This study was approved by the chairs of the different departments of Internal Medicine of the University Medical Center Utrecht. The ethical commission of the University Medical Center Utrecht approved institutional review board exemption, since this was a retrospective study and participants were not subject to procedures or were not required to follow rules of behaviour. For personal data protection, all data were anonymized before analysis. This study was performed according to the Declaration of Helsinki. We followed patients for three months, until an outcome event (bleeding, thrombosis (venous thrombosis or ischemic stroke) or death) or the end of the follow-up period (June 2019).

### Low molecular weight heparin guideline

Our local clinical protocol follows the national Dutch guideline which recommends a dosage reduction of LMWH in patients with an eGFR below 60 ml/min/1.73m^2^, followed by an anti-Xa level measurement when LMWH is continued for more than three days [13]. Dalteparin is the most prescribed LMWH for therapeutic indications in our center followed by nadroparin. A dosage reduction of 25% for patients with an eGFR between 30 and 60 ml/min/1.73m^2^ and of 50% for patients with an eGFR <30 ml/min/1.73m^2^ is recommended after a full first dose of 100%. The recommended dosage per subcutaneous injection is 200 international units (IU) per kg for once daily dalteparin and 100 IU/kg for twice daily. The recommended dosage per subcutaneous injection is 171 IU/kg for once daily nadroparin and 86 IU/kg for twice daily. We categorized patients into three groups (dosage below, according or above the guideline recommendations) based on weight and eGFR according to the guideline and local protocol.

### Anti-Xa measurements

Anti-Xa levels were measured four hours after LMWH administration on the third day of LMWH use for both patient with once and twice daily LMWH use. Blood samples were collected in sodium citrate tubes (3.2%, 0.105 M, BD vacutainer, Becton Dickinson, UK). All laboratory measurements were performed in the central clinical ISO15189 certified laboratory of the University Medical Center Utrecht Utrecht. Until June 2018, anti-Xa levels were measured on a STA-Rack evolution coagulation analyser using STA liquid anti-Xa reagent (Diagnostica Stago, Asnières-sur-Seine, France). From June 2018, anti-Xa levels were measured on an ACL Top 750 LAS coagulation analyser using HemoSiL Liquid Anti-Xa reagent (Werfen, Bedford, MA, USA). Both anti-Xa assays were carefully aligned. All assays participated in robust internal and external quality assessment schemes and local performance characteristics were within the pre-defined limits stated by the manufacturers.

The target range for peak anti-Xa levels was between 1.0 U/ml and 2.0 U/ml with once daily LMWH and was between 0.6 U/ml and 1.0 U/ml with twice daily LMWH according to our local protocol and the Dutch guideline. Based on this target range, patients had anti-Xa levels below the range if anti-Xa levels were <1.0 U/ml for once daily LMWH and <0.6 U/ml for twice daily LMWH and above range if anti-Xa levels were >2.0 U/ml for once daily LMWH and >1.0 U/ml for twice daily LMWH.

### Demographic and clinical data

Demographic and clinical data were obtained from the electronic medical record. Baseline was set as the day of the first anti-Xa measurement. Data on age, sex, height, weight, LMWH (dalteparin or nadroparin), dosage, treatment frequency, antiplatelet drug use, indication for anticoagulation, prior ischemic stroke, prior bleeding, malignancy, liver cirrhosis, heart failure, hypertension history, peripheral vascular disease, hemoglobin and plasma creatinine concentrations were collected at baseline. The creatinine value preceding the anti-Xa measurement was used to calculate eGFR using the CKD-EPI formula [14]. In 94% of the patients, creatinine measurements were performed within three days before the anti-Xa measurement.

### Study outcomes: Bleeding, thrombosis and mortality

Endpoints of the study were major bleeding, thrombotic events (ischemic stroke or venous thrombosis including deep vein thrombosis or pulmonary embolism) and mortality within three months after baseline (date of anti-Xa measurement). Major bleeding events were collected from the medical records and evaluated following the classification of the Scientific and Standardization Committee of the International Society on Thrombosis and Haemostasis [15]. A major bleeding was defined as a fatal bleeding or a symptomatic bleeding in a critical area or organ (intracranial, intraspinal, intraocular, retroperitoneal, intra‐articular, pericardial, or intramuscular with compartment syndrome) or bleeding causing a fall in hemoglobin level of more than 20 g/L (1.24 mmol/L) or a bleeding event leading to transfusion of two or more units of red cells. Venous thrombosis was confirmed by compression ultrasound for deep vein thrombosis of the leg or arm. Furthermore, pulmonary embolism had to be diagnosed by computed tomography. Ischemic stroke was confirmed by computed tomography or magnetic resonance imaging.

### Statistical analyses

Continuous variables are presented as median and interquartile rage (IQR). Categorical variables are presented as counts with corresponding percentages.

To investigate the association between dosage reduction of LMWHs and anti-Xa levels, we calculated the proportions of anti-Xa levels that fell within the range across different groups of guideline adherence (dosage below, within and above guideline recommendation). These analyses were stratified for different eGFR categories (eGFR between 30 and 60 ml/min/1.73m^2^ and eGFR <30 ml/min/1.73m^2^). Furthermore, we stratified these analyses for dalteparin and nadroparin use.

The observation time in each participant was calculated as the time elapsed between the date of the first anti-Xa level measurement and the outcome event within three months (bleeding, thrombosis or mortality) or censoring date (June 2019). Incidence rates for bleeding, thrombosis or mortality were estimated by dividing the number of patients with an event by the total observation time at risk. Only the first event was taken into account. When estimating the incidence rates for bleeding, we neglected the occurrence of thrombosis and vice versa.

In addition, we calculated crude and adjusted hazard ratios (HRs) with 95% confidence intervals (CIs) to evaluate the association between anti-Xa level categories (below, within and above target range) and outcomes (bleeding, thrombosis and mortality within three months). Hazard ratios were adjusted for age, sex, body mass index, LMWH (dalteparin or nadroparin), daily frequency of LMWH dosing (once or twice daily), antiplatelet drug use, indication for anticoagulation, prior ischemic stroke, prior bleeding, malignancy, liver cirrhosis, heart failure, hypertension history, peripheral vascular disease, hemoglobin concentration and eGFR. Furthermore, we investigated the association between outcomes (bleeding, thrombosis and mortality within three months) and a dosage of LMWH below or above the guideline recommendation as compared with a dosage according to the guideline.

As a sensitivity analysis, a time-dependent cox regression analysis was performed to account for potential influence of changes in anti-Xa levels over time. In addition, we performed a competing risk analysis to calculate hazard ratios for bleeding and thrombosis accounting for death as a competing risk. Furthermore, we also investigated the association between anti-Xa levels (below, within and above target range) and outcomes (major bleeding, thrombosis and mortality) within one month. Finally, we also investigated the association between LWMH dosing and anti-Xa levels and outcomes excluding patients from June 2018 (n = 39), since anti-Xa levels were measured with another analyser. All analyses were done by use of SPSS statistical software version 25.0 (IBM SPSS Statistics).

## Results

### Baseline characteristics

**Fig 1:**
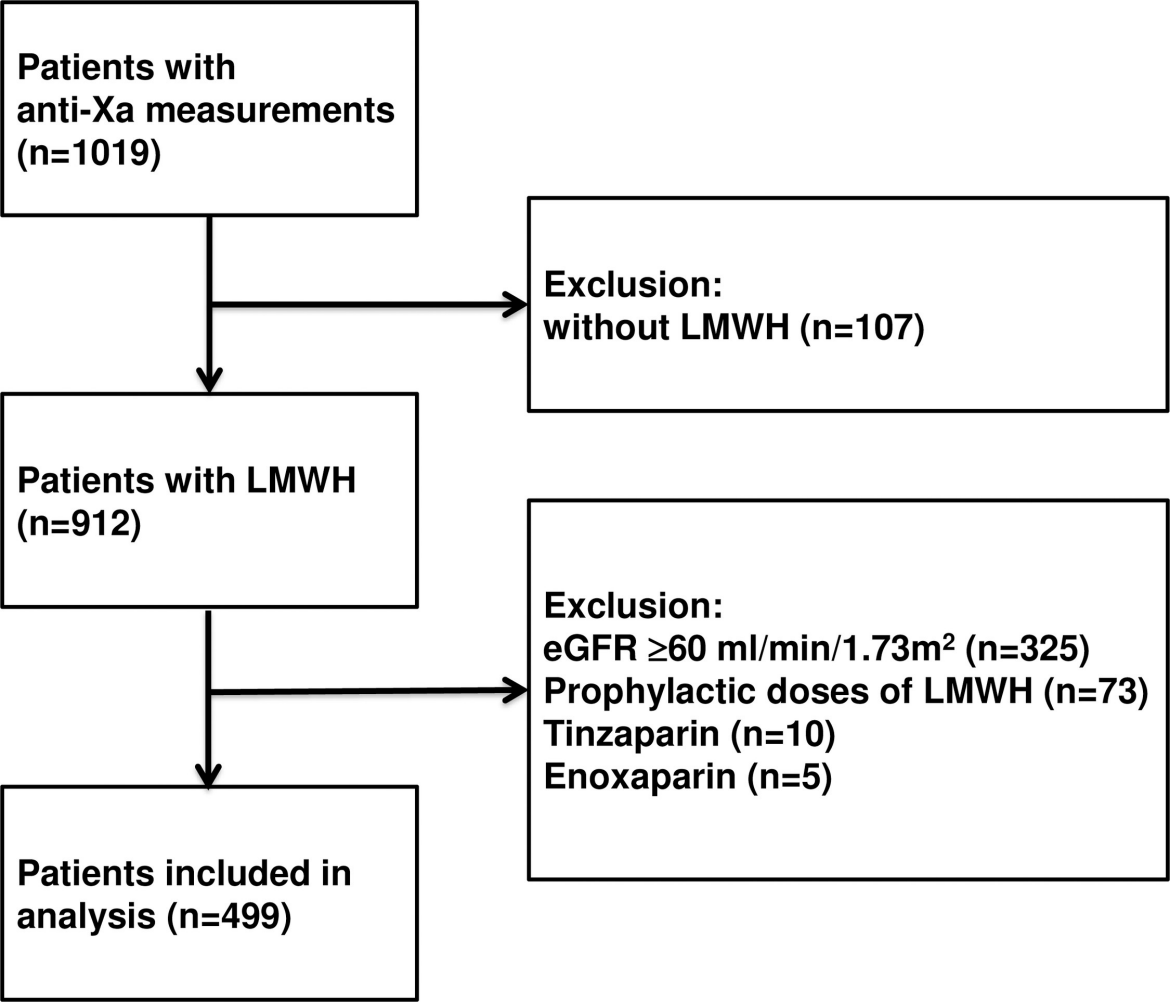
Study outline.

We included 499 adult patients with an anti-Xa measurement and therapeutic use of dalteparin or nadroparin in our analysis. Patients with an eGFR >60 ml/min/1.73m^2^ (n = 325), who did not use LMWHs (n = 107), with prophylactic dosages of LMWH (n = 73), or patients who used tinzaparin (n = 10) or enoxaparin (n = 5) were excluded (Fig 1).

The median age of patients was 68 years and 39% of the included patients were women (Table 1). Dalteparin was used by 89% of the patients, while nadroparin was used in 11% of the patients. Indication for LMWH use was venous thrombosis in 45%, atrial fibrillation in 37%, artificial heart valves in 7% and 11% of the patient had other reasons. Of the 499 patients, 42% had an eGFR between 30 and 60 ml/min/1.73m^2^ (median eGFR 43 ml/min/1.73m^2^) and 58% had an eGFR below 30 ml/min/1.73m^2^ (median eGFR 17 ml/min/1.73m^2^). There were 78 dialysis patients in our study (69 hemodialysis and 9 peritoneal dialysis patients).

**Table 1:** Baseline characteristics.

			Total (n = 499)
Age, median (IQR), years			68 (60–77)
Sex female (%)			195 (39%)
Low molecular weight heparin			
		Dalteparin once daily	163 (33%)
		Dalteparin twice daily	282 (57%)
		Nadroparin once daily	15 (3%)
		Nadroparin twice daily	39 (8%)
Antiplatelet drug use			87 (17%)
Indication for anticoagulation			
		Atrial fibrillation	223 (45%)
		Venous thrombosis	182 (37%)
		Prosthetic heart valves	37 (7%)
		Other	57 (11%)
Comorbidities			
		Prior ischemic stroke	49 (10%)
		Prior bleeding	169 (34%)
		Malignancy	200 (40%)
		Liver cirrhosis	14 (3%)
		Heart failure	74 (15%)
		Hypertension history	348 (70%)
		Peripheral vascular disease	85 (17%)
Weight, median (IQR), kg			78 (68–90)
Body mass index, median (IQR), kg/m2			25.6 (22.9–29.4)
Hemoglobin, median (IQR), g/L			9.8 (8.9–11.0)
Renal function (eGFR), median (IQR), ml/min/1.73m^2^			27 (15–41)
	30–60 ml/min/1.73m^2^		212 (42%)
	<30 ml/min/1.73m^2^		287 (58%)

IQR, interquartile rage.

### Anti-Xa levels

The median anti-Xa levels were 0.44 U/ml (IQR 0.21–0.75) for the total group (n = 499), 0.45 U/ml (IQR 0.17–0.70) for once daily dalteparin users (n = 163), 0.40 U/ml (IQR 0.21–0.64) for twice daily dalteparin users (n = 282), 1.15 U/ml (IQR 0.95–1.48) for once daily nadroparin users (n = 15) and 0.59 U/ml (IQR 0.28–0.92) for twice daily nadroparin users (n = 39).

Table 2 shows the proportion of patients who had anti-Xa levels below, within and above the target range. In the total group, only 21% of the patients had a first anti-Xa measurement within range, while 73% of the patients were below the target range and 6% of the patients were above the target range. Declining eGFR resulted in increasing proportion of patients below the target range. An eGFR between 30 and 60 ml/min/1.73m^2^ resulted in 66% of the patients below target range and an eGFR below 30 ml/min/1.73m^2^ resulted in 78% of the patients below target range. In the dialysis group, only 14% of the patients had a first anti-Xa measurement within range, while 78% of the patients were below the target range and 8% of the patients were above the target range.

**Table 2:** Distribution of anti-Xa levels.

Anti-Xa levels*				Total (n = 499)	Dosage below guideline (n = 46)	Dosage according guideline (n = 178)	Dosage above guideline (n = 275)
**eGFR <60 ml/min/1.73m**^**2**^							
	Below range			363 (73%)	42 (91%)	138 (78%)	183 (67%)
	Within range			104 (21%)	4 (9%)	33 (19%)	67 (24%)
	Above range			32 (6%)	0 (0%)	7 (4%)	25 (9%)
		**eGFR 30–60 ml/min/1.73m**^**2**^					
			Below range	140 (66%)	41 (91%)	67 (70%)	32 (45%)
			Within range	56 (26%)	4 (9%)	23 (24%)	29 (41%)
			Above range	16 (8%)	0 (0%)	6 (6%)	10 (14%)
		**eGFR <30 ml/min/1.73m**^**2**^					
			Below range	223 (78%)	1 (100%)	71 (87%)	151 (74%)
			Within range	48 (17%)	0 (0%)	10 (12%)	38 (19%)
			Above range	16 (6%)	0 (0%)	1 (1%)	15 (7%)

*Anti-Xa levels: below range (<1.0 U/ml for once daily low molecular weight heparin and <0.6 U/ml for twice daily low molecular weight heparin), within range (1.0 U/ml—2.0 U/ml for once daily low molecular weight heparin and 0.6 U/ml—1.0 U/ml for twice daily low molecular weight heparin), above range (>2.0 U/ml for once daily low molecular weight heparin and >1.0 U/ml for twice daily low molecular weight heparin).

Overall, only 19% of the patients with an eGFR <60 ml/min/1.73m^2^ who received a dosage according to the guideline had anti-Xa levels within the target range. The great majority (91%) of patients with a dosage below guideline recommendation had anti-Xa levels below the target range. Moreover, a dosage above the current guideline advice still resulted in anti-Xa levels that were below the target range in 67% of the total cohort.

Table 3 displays differences in the anti-Xa levels between dalteparin and nadroparin users. A dosage according to the guideline resulted in anti-Xa levels within the target range for 12% of the dalteparin users and for 50% of the nadroparin users. A dosage according to the guideline resulted in 2% of the patients within the anti-Xa target range for once daily dalteparin use and 16% for twice daily use. For nadroparin use, 73% of the patients with once daily use and 38% of the patients with twice daily use had an anti-Xa level within target range with a dosage according to the guideline.

**Table 3:** Distribution of anti-Xa levels stratified for dalteparin and nadroparin.

				DALTEPARIN			NADROPARIN		
anti-Xa levels*				Dosage below guideline (n = 33)	Dosage according guideline (n = 146)	Dosage above guideline (n = 264)	Dosage below guideline (n = 13)	Dosage according guideline (n = 32)	Dosage above guideline (n = 9)
**eGFR <60 ml/min/1.73m**^**2**^									
	Below range			31 (94%)	126 (86%)	182 (68%)	11 (85%)	12 (38%)	1 (11%)
	Within range			2 (6%)	17 (12%)	64 (24%)	2 (15%)	16 (50%)	3 (33%)
	Above range			0 (0%)	3 (2%)	20 (8%)	0 (0%)	4 (13%)	5 (56%)
		**eGFR 30–60 ml/min/1.73m**^**2**^							
			Below range	30 (94%)	64 (78%)	32 (48%)	11 (85%)	3 (21%)	0 (0%)
			Within range	2 (6%)	16 (20%)	26 (39%)	2 (15%)	7 (50%)	3 (75%)
			Above range	0 (0%)	2 (2%)	9 (13%)	0 (0%)	4 (29%)	1 (25%)
		**eGFR <30 ml/min/1.73m**^**2**^							
			Below range	1 (100%)	62 (97%)	150 (75%)	0 (0%)	9 (50%)	1 (20%)
			Within range	0 (0%)	1 (2%)	38 (19%)	0 (0%)	9 (50%)	0 (0%)
			Above range	0 (0%)	1 (2%)	11 (6%)	0 (0%)	0 (0%)	4 (80%)

*Anti-Xa levels: below range (<1.0 U/ml for once daily low molecular weight heparin and <0.6 U/ml for twice daily low molecular weight heparin), within range (1.0 U/ml—2.0 U/ml for once daily low molecular weight heparin and 0.6 U/ml—1.0 U/ml for twice daily low molecular weight heparin), above range (>2.0 U/ml for once daily low molecular weight heparin and >1.0 U/ml for twice daily low molecular weight heparin).

A second measurement of anti-Xa levels was done within one week after the first measurement in 171 (47%) of the 363 patients with anti-Xa levels below range in the first measurement. For these 171 patients, the second anti-Xa level was still below range in 71%, within range in 26% and above range in 3%. For the 32 patients who had a first anti-Xa level above range, a second measurement was done in 12 patients within one week (38%). In these 12 patients, the second anti-Xa level was below range in 17%, within range in 42% and still above range in 42%.

### Bleeding

There were 104 first major bleeding events within 3 months of follow-up, of which 26 were intracranial and 78 were extracranial bleeding events. Of the 104 major bleeding events, 20 were fatal bleedings. The major bleeding rate within three months of follow-up was 1238 major bleeding events per 1000 person-years. There was no relation with anti-Xa levels below or above the target range and bleeding events (Table 4). A dosage below (HR 0.9, 95% CI 0.4–2.0) or above (HR 0.9, 95% CI 0.6–1.5) the guideline recommendation as compared with a dosage according to the guideline was not associated with major bleedings after adjustment.

**Table 4:** Anti-Xa levels and bleeding, thrombosis and mortality within three months.

		BLEEDING		THROMBOSIS		MORTALITY	
Anti-Xa levels*		Crude HR	Adjusted**	Crude	Adjusted**	Crude	Adjusted**
		(95% CI)	HR (95% CI)	HR (95% CI)	HR (95% CI)	HR (95% CI)	HR (95% CI)
**eGFR <60 ml/min/1.73m**^**2**^							
	Below range	1.1 (0.7–1.8)	1.0 (0.6–1.8)	2.4 (0.7–7.9)	2.5 (0.7–8.8)	1.2 (0.8–1.8)	1.2(0.8–1.9)
	Within range	1 ref	1 ref	1 ref	1 ref	1 Ref	1 ref
	Above range	1.2 (0.5–2.8)	1.4 (0.6–3.4)	NA	NA	0.8 (0.4–1.9)	1.0 (0.4–2.4)

NA, not applicable.

*Anti-Xa levels: below range (<1.0 U/ml for once daily low molecular weight heparin and <0.6 U/ml for twice daily low molecular weight heparin), within range (1.0 U/ml—2.0 U/ml for once daily low molecular weight heparin and 0.6 U/ml—1.0 U/ml for twice daily low molecular weight heparin), above range (>2.0 U/ml for once daily low molecular weight heparin and >1.0 U/ml for twice daily low molecular weight heparin).

**Adjusted for age, sex, body mass index, low molecular weight heparin (dalteparin or nadroparin), daily frequency of low molecular weight heparin dosing (once or twice daily), antiplatelet drug use, indication for anticoagulation, prior ischemic stroke, prior bleeding, malignancy, liver cirrhosis, heart failure, hypertension history, peripheral vascular disease, hemoglobin levels and eGFR.

### Thrombosis

There were 27 first thrombotic events (7 patients with ischemic stroke and 20 patients with venous thrombosis) corresponding to an event rate of 281 thrombotic events per 1000 person-years. Of the 27 thrombotic events, 8 were fatal. Anti-Xa level below the target range as compared with adequate anti-Xa levels were associated with a 2.4-fold (95% CI 0.7–7.9) increased thrombotic risk, which did not materially change after adjustment for age, sex, body mass index, LMWH (dalteparin or nadroparin), daily frequency of LMWH dosing (once or twice daily), antiplatelet drug use, indication for anticoagulation, prior ischemic stroke, prior bleeding, malignancy, liver cirrhosis, heart failure, hypertension history, peripheral vascular disease, hemoglobin levels and eGFR (HR 2.5, 95% CI (0.7–8.8)) (Table 4).

The hazard ratio of thrombosis was 2.5 (95% CI 0.7–9.0) for a dosage below the guideline recommendation as compared with a dosage according to the guideline after adjustment. A dosage above the guideline recommendation was not associated with thrombotic events (HR 1.0, 95% CI 0.4–2.7).

### Mortality

Of the 499 patients, 146 patients died within three months. The mortality rate was 1471 per 1000 person-years. We did not find an association between anti-Xa levels and mortality (Table 4).

A dosage below (HR 1.0, 95% CI 0.6–1.8) or above (HR 0.8, 95% CI 0.5–1.1) the guideline recommendation as compared with a dosage according to the guideline was not associated with mortality after adjustment.

### Sensitivity analyses

The time-dependent cox regression analyses showed similar results as the analysis in which only the first anti-Xa measurement was taken into account. There was no relation with anti-Xa levels above the target range and bleeding events after adjustments (HR 1.0, 95% CI 0.4–2.4). Furthermore, time-dependent cox regression analysis showed an adjusted HR of 1.6 (95% CI 0.6–4.4) of thrombosis for anti-Xa level below the target range as compared with adequate anti-Xa levels. We did not find an association between anti-Xa levels and mortality.

Competing risk models accounting for death showed also similar associations regarding the risk of bleeding and thrombosis. There was also no relation with anti-Xa levels above the target range and bleeding events after adjustments (HR 1.1, 95% CI 0.6–1.8) when accounting death as competing risk. Furthermore, HR of thrombosis was 2.5 (95% CI 0.6–9.6) for anti-Xa level below the target range as compared with adequate anti-Xa levels.

We also investigated the association between anti-Xa levels and outcomes within one month (Table 5). These hazard ratios for one month were comparable with the hazard ratios for three months.

**Table 5:** Anti-Xa levels and bleeding, thrombosis and mortality within one month.

		BLEEDING	THROMBOSIS	MORTALITY
Anti-Xa levels		Adjusted*	Adjusted*	Adjusted*
		HR (95% CI)	HR (95% CI)	HR (95% CI)
**eGFR <60 ml/min/1.73m**^**2**^				
	Below range	1.1 (0.6–1.9)	3.3 (0.7–15.2)	1.1 (0.6–1.9)
	Within range	1 ref	1 ref	1 ref
	Above range	1.7 (0.7–4.6)	NA	1.3 (0.5–3.3)

NA, not applicable.

*Adjusted for age, sex, body mass index, low molecular weight heparin (dalteparin or nadroparin), daily frequency of low molecular weight heparin dosing (once or twice daily), antiplatelet drug use, indication for anticoagulation, prior ischemic stroke, prior bleeding, malignancy, liver cirrhosis, heart failure, hypertension history, peripheral vascular disease, hemoglobin levels and eGFR.

Furthermore, we investigated whether results were different when patients were excluded after June 2018 (n = 39), since anti-Xa levels were measured with another analyser. However, this did not change the results. In the total group, a similar percentage of only 21% of the patients had a first anti-Xa measurement within range. In addition, we did not find an association between anti-Xa levels and bleeding, thrombosis or mortality.

## Discussion

In this observational cohort study of 499 patients with decreased renal function who received therapeutic dosages of nadroparin or dalteparin, we showed that a pre-emptive dosage reduction of LMWH (25% reduction in patients with an eGFR between 30 and 60 ml/min/1.73m^2^ and a reduction of 50% in in patients with an eGFR below the 30 ml/min/1.73m^2^) leads to adequate levels of anti-Xa in only 19% of the patients. Furthermore, there were large differences in the proportion of patients within the target ranges for dalteparin and nadroparin use. Dosage reduction according to the guideline for patients with eGFR below 60 ml/min/1.73m^2^ resulted in adequate anti-Xa levels in 50% of the nadroparin users and in 12% of the dalteparin users. We did not find an association between anti-Xa levels and bleeding, thrombosis or mortality.

Several previous studies have reported on the association between dosages of LMWHs and anti-Xa levels, mostly focussing on enoxaparin [8, 16–21]. Unadjusted therapeutic dosages of enoxaparin were associated with increased anti-Xa levels in patients with an eGFR <30 ml/min/1.73m^2^ as compared with patients with an eGFR>30 ml/min/1.73m^2^ [8, 20, 21]. Furthermore, several observational studies reported that dosage reduction of enoxaparin in patients with a decreased kidney function led to adequate anti-Xa levels [16–19]. Therefore, these studies recommended pre-emptive dose reductions of enoxaparin in patients with decreased kidney function. In contrast to enoxaparin, previous studies did not find an association between decreased kidney function and increased anti-Xa levels for tinzaparin [22–25].

Few studies have reported on the association between nadroparin or dalteparin use and anti-Xa levels. A previous study showed that anti-Xa activity in patients with eGFR below 60 ml/min/1.73 m^2^ treated with pre-emptive dosage reduction of therapeutic dosages of nadroparin resulted in 51% of the anti-Xa levels within the target range, which is similar to our results [26]. To our knowledge, there are no studies that investigated the association between anti-Xa levels and dalteparin for unadjusted and adjusted dosages in patients with impaired kidney function. Dalteparin (6000 Dalton) has a similar molecular weight as tinzaparin (6500 Dalton) and has a higher molecular weight than nadroparin (4300 Dalton) or enoxaparin (4500 Dalton) [27, 28]. Our finding that a dosage reduction in dalteparin for patients with eGFR below the 60 ml/min/1.73m^2^ resulted in adequate anti-Xa levels in 12% of the patients was in line with the finding of a previous study that showed adequate anti-Xa levels in only 8% of tinzaparin users [25].

The greater proportion of anti-Xa levels within target range for nadroparin (50%) than with dalteparin (12%) after pre-emptive dose reduction in patients with an eGFR <60 ml/min/1.73m^2^ can be explained by the difference in molecular weight (4300 Dalton versus 6000 Dalton, respectively) [29]. LMWHs are cleared through a combination of depolymerization after binding to endothelial cell receptors and macrophages and renal filtration. The degree of renal clearance of LMWHs are dependent on the renal function and on the molecular weight [12]. Therefore, the lower molecular weight of nadroparin than dalteparin leads to a longer biological half-life of nadroparin than dalteparin. Consequently, a strategy of pre-emptive dosage reduction of LMWHs probably leads to lower anti-Xa levels of dalteparin in comparison with nadroparin.

Previous studies also investigated the association between anti-Xa levels and outcome events [30–34]. Low anti-Xa levels (<0.5 IU/ml) as compared with anti-Xa levels in the range of 0.5 to 1.2 IU/ml were found to be associated with an increased 30-day mortality risk [30]. We did not find an association between anti-Xa levels and mortality in our study. Another study in patients after hip replacement reported that low anti-Xa levels were associated with increased post-operative thrombosis risk, while high anti-Xa levels were associated with increased bleeding (wound hematoma) [31]. Yet, another study in patients with acute venous thromboembolism who were treated with dalteparin demonstrated that increased anti-Xa levels (above 0.8 IU/ml) were associated with increased bleeding risks [32]. In line with our study, two previous studies did not show an association between anti-Xa levels and bleeding [33, 34].

The strengths of the present study are the large number of participants with information on anti-Xa levels, kidney function and information on bleeding and thrombotic events. To our knowledge, this is the first study that evaluated the association between dosage reduction of dalteparin and nadroparin, anti-Xa levels and outcomes (bleeding, thrombosis and mortality). This study has also several limitations. First, patients who were treated with tinzaparin and enoxaparin were excluded because of the low number of patients. Second, patients from our tertiary center in our study reflect a high-risk group with increased bleeding, thrombosis and mortality rates in which anti-Xa levels were measured. Therefore, it could be that our findings are not generalizable to all patients with renal dysfunction treated with LMWHs. Third, only in a small proportion of patients a second measurement of anti-Xa levels was performed. This could be a selected group in which a low anti-Xa level was expected explaining the low levels in the second anti-Xa level measuring. Fourth, we had limited power in the group of dialysis patients, patients with a LMWH dosage lower than the recommended dose and in the group of patients who used nadroparin. Furthermore, confidence intervals for thrombotic risk were very wide, because of a limited powered due to the low number of thrombotic events. Fifth, the cause of death was not registered. Sixth, we have no data about the time of LMWH administration and time of blood drawing. Therefore, we cannot exclude the possibility of incorrect drawing leading to low peak target ranges. Finally, we cannot exclude residual confounding and selection bias in the association between anti-Xa levels and outcomes. Selection could have occurred in different stages of our study. First, anti-Xa levels are probably not measured in all patients with a an eGFR below 60 ml/min/1.73m^2^ who used LMWH. Second, it is not clear which patients continued oral anticoagulants and which patients were switched to LMWHs during hospitalization. Third, differences in anti-Xa levels between once versus twice daily dosing of LMWH and nadroparin versus dalteparin use could be due to selection bias rather than pharmacological differences.

The American guideline “Parenteral Anticoagulants: American College of Chest Physicians Evidence Based Clinical Practice Guidelines (9th Edition)” and the Dutch guideline “Anticoagulation with LMWHs in renal impairment” suggests anti-Xa monitoring and dose reductions in patients with renal impairment [12, 13]. The American College of Chest Physicians Evidence Based Clinical Practice Guidelines suggestion for anti-Xa monitoring is made in a summary chapter that does not provide graded recommendations. The Dutch guideline is based on expert opinion. In contrast, the American Society of Hematology (ASH) guideline panel for optimal management of anticoagulation therapy for venous thromboembolism makes a graded recommendation against anti-Xa monitoring in patients with an eGFR below 30 ml/min/1.73m^2^ receiving LMWH for treatment of venous thrombo-embolism (conditional recommendation based on very low certainty in the evidence about effects) [35].

Although the rationale of dosage reductions and anti-Xa monitoring of LMWHs is clear, we showed no association between anti-Xa levels and outcomes. Therefore, our data supports the ASH recommendation against anti-factor Xa monitoring. Moreover, there are some other problems with anti-Xa monitoring. It is not known whether measurements of anti-Xa levels must be performed after each dosage adjustment and whether anti-Xa level monitoring should be continued in patients who are within range. Based on the poor performance of the Dutch Federation of Nephrology guideline recommendation in clinical practice, future studies should investigate whether a strategy without anti-Xa measurements as suggested by the ASH guideline is non-inferior to an anti-Xa guided dosing strategy in patients with an impaired kidney function as suggested by the Dutch and American College of Chest Physicians Evidence Based Clinical Practice guideline.

In conclusion, pre-emptive dosage reduction of LMWHs in patient with an eGFR <60 ml/min/1.73m^2^ leads to a high proportion of patient below target anti-Xa ranges. However, low anti-Xa levels were not associated with clinical outcomes in our study.
